# Reconstruction of a Large, Central Forehead Defect After Mohs Micrographic Surgery Using the Innovative Horizontal Double‐Banner Transposition “Buffalo‐Flap” Technique

**DOI:** 10.1155/crot/2928920

**Published:** 2026-04-13

**Authors:** Mojahed Mohammad K. Shalabi, Mohamad Jabin, Marcus Zaayman, Wasiq Nadeem, Matthew K. Lee, Chad Housewright

**Affiliations:** ^1^ Department of Dermatology, Baylor Scott & White Medical Center, Temple, Texas, USA; ^2^ Texas A&M School of Medicine, Dallas, Texas, USA; ^3^ Division of Otolaryngology-Head and Neck Surgery, Cedars-Sinai Medical Center, Los Angeles, California, USA, cedars-sinai.edu; ^4^ Keck School of Medicine, University of Southern California, Los Angeles, California, USA, usc.edu

**Keywords:** banner transposition flap, buffalo flap, flap reconstruction, large forehead defect, Mohs micrographic surgery

## Abstract

Forehead reconstruction after surgical management of cutaneous malignancy is dependent on the depth and location of the wound. In addition, preserving the symmetry and position of the eyebrow and eyelids is key to achieving a favorable cosmetic outcome, specifically avoiding an unintentional elevation of the brow which can result in an unfavorable “surprised” appearance. Appropriate patient selection is also critical, with skin laxity and patient preference playing a role. The recently proposed “Buffalo flap” is a promising treatment option for the reconstruction of large, central forehead defects in elderly patients in whom bone is exposed.

## 1. Introduction

**FIGURE 1:**
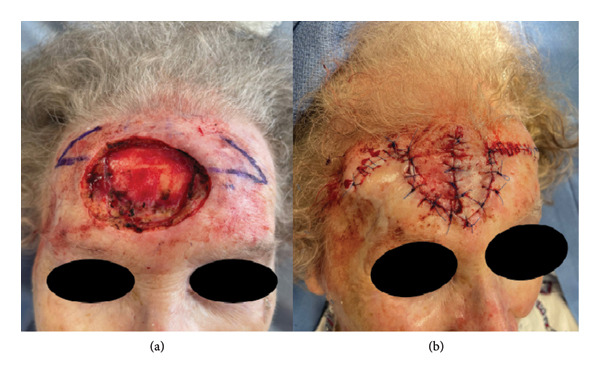
(a) Defect after Mohs micrographic surgery for recurrent squamous cell carcinoma of the central forehead along with the design of the two horizontal banner flaps that resembles that of a buffalo head. (b) Immediate postoperative result.

Forehead reconstruction options following malignancy resection are numerous; these options include primary closure, skin graft, unilateral or bilateral advancement flaps, rhombic transposition flap, and banner transposition flap [1, 2]. However, when the defect extends to bone, as in our case, local flaps are the preferred reconstruction option, as these will achieve better contour when compared to skin grafting techniques and can also be utilized even if the periosteum is absent [3]. The “Buffalo flap,” originally described by Andreas Skaria in 2021, is a horizontal double‐banner transposition flap that allows for the coverage of large, central forehead defects without causing asymmetry to the eyebrow line [1]. While named as such in 2021, it is important to note that similar symmetric bilateral transposition flaps have been described in the literature. For such defects, symmetry is critical to achieving optimal esthetic outcomes in midline reconstructions, and a similar philosophy of using bilateral flaps with opposing vectors has been described for midline defects of the nasal tip [4]. This unique flap can result in a favorable functional and cosmetic outcome in large, central forehead calvarial defects (Figure 1), with important considerations given to certain limitations.

## 2. Discussion

Reconstruction options for forehead defects vary depending on the elasticity of the skin and the size, depth, and laterality of the defect. Different approaches to forehead flap reconstruction have been published in the literature. Primary closures and skin grafts are suitable for small to medium forehead defects that do not expose the calvarium [3]. For large, midline forehead defects with bone exposure, linear advancement flaps such as a bilateral O‐H flap, otherwise known as H‐plasty, have been demonstrated to be effective repair techniques but have the cosmetic downsides of multiple long incision lines required to recruit adjacent tissue from two ends [1, 2]. The A‐T advancement flap is mainly utilized in lateral defects where donor tissue is recruited from the temple; however, this repair would not be appropriate in our case given the defect location [1]. In certain cases, especially in the setting of exposed bony defects of the scalp, staged skin grafting may also be an option in which the outer cortex may be burred, followed by the placement of a temporary xenograft with eventual delayed split‐thickness skin grafting. While the “Buffalo flap” was described and named as such in 2021, it is important to note that similar types of reconstruction have been reported in the literature, effectively using bilateral and symmetric transposition flaps to close large defects [5, 6]. Other similar defects have been repaired using a sling island pedicle for a similar defect [7]. For flap reconstruction on the forehead, numbness is common postoperatively given the significant amount of adjacent tissue transfer in large defects [8].

**FIGURE 2:**
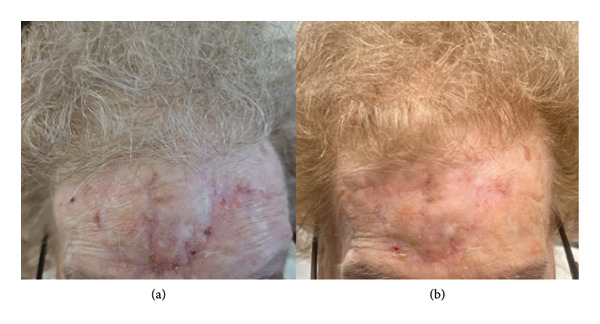
(a) Two‐month wound check visit after reconstructive surgery. (b) Five‐month wound check visit after reconstructive surgery.

Forehead reconstruction after surgical management of cutaneous malignancy is dependent on the depth and location of the wound [1, 2]. Additionally, the forehead is an area of relatively lower laxity, which may make such a flap more difficult to perform. In this case, the patient was elderly with substantial skin laxity, which allowed for a successful and esthetically fair flap outcome (Figure 2). In patients with less laxity, as is generally the case on the forehead, bilateral transposition flaps may cause extreme tension, increasing the risk for flap necrosis and delayed healing. In addition, preserving the symmetry and position of the eyebrow and eyelids is key to achieving a favorable cosmetic outcome, specifically avoiding an unintentional elevation of the brow which can result in an unfavorable “surprised” appearance [2]. Further drawbacks to this reconstruction include the presence of three geometric, vertical scar lines in the mid‐frontal area in the initial months of healing and vascular fragility of the flap tip. While not the case in our patient, these flaps may also lead to a pincushion defect, which may need to be addressed down the line by way of steroid injections or surgical debulking. It is of utmost importance to note that appropriate patient selection is critical for such a flap, with great attention given to skin laxity and the location of the defect. As such, this flap may be a good option in select patients with skin laxity and those who may want to avoid staged procedures or skin grafts. The “Buffalo flap” serves as a useful tool in the armamentarium for reconstructive surgeons and should be considered in appropriate patients.

## Funding

This article has no funding source.

## Disclosure

None of the contents herein have been published previously nor are they under consideration for publication elsewhere.

## Consent

Patient consent was obtained verbally for publication.

## Conflicts of Interest

The authors declare no conflicts of interest.

## Data Availability

The data that support the findings of this study are available from the corresponding author upon reasonable request.

## References

[1] Skaria A. M. , Reconstruction of a Large Central Forehead Defect, Dermatologic Surgery. (2021) 47, no. 12, 1619–1621, 10.1097/DSS.0000000000002721. 33625148

[2] Hankinson A. and Holmes T. , Repair of Defects of the Central Forehead With a Modified Banner Transposition Flap, Dermatologic Surgery. (2018) 44, no. 3, 459–462, 10.1097/DSS.0000000000001318, 2-s2.0-85046858153. 28902032

[3] Shimizu R. and Kishi K. , Skin Graft, Plastic Surgery International. (2012) 2012, 563493–563495, 10.1155/2012/563493. 22570780 PMC3335647

[4] Baird B. J. , Moubayed S. P. , and Most S. P. , A Comparison of the Double-Half Bilobe Flap to the Traditional Bilobe Flap: Cohort Analysis of a Single Surgeon Experience, Facial Plastic Surgery. (2017) 33, no. 5, 526–529, 10.1055/s-0037-1606333, 2-s2.0-85030231344. 28962059

[5] Verdolini R. , Malik M. , and Hirbod T. , Bilateral Symmetric Transposition Flap (Pincer Flap) for Reconstruction of a 19-cm^2^ Defect of the Forehead, European Journal of Dermatology. (2016) 26, no. 6, 626–627, 10.1684/ejd.2016.2862, 2-s2.0-85007418219. 27707669

[6] Freeman S. C. , Neill B. , Garvey C. , and Leitenberger J. J. , Bilateral Transposition Flaps With Split-Thickness Skin Grafting of Secondary Defects After a Large Mohs Micrographic Surgery Defect With Exposed Calvarium, Cureus. (2023) 15, no. 7, 10.7759/cureus.42191. PMC10439768 37602082

[7] Neill B. C. , Siscos S. M. , Seger E. W. , and Hocker T. L. H. , Reconstruction of a Large Upper Paramedian Forehead Defect, Dermatologic Surgery. (2023) 49, no. 1, 93–95, 10.1097/DSS.0000000000003464. 35522552

[8] Lutz M. E. , Otley C. C. , Roenigk R. K. , Brodland D. G. , and Li H. , Reinnervation of Flaps and Grafts of the Face, Archives of Dermatology. (1998) 134, no. 10, 1271–1274, 10.1001/archderm.134.10.1271, 2-s2.0-0031763294. 9801683

